# Promoting evolution: the brand new *Hellenic Evolutionary Society (HEVOS)*

**DOI:** 10.1186/s40709-019-0098-6

**Published:** 2019-08-30

**Authors:** Theodore J. Abatzopoulos, Sinos Giokas, Panayiotis Pafilis, Nikos Poulakakis, Spyros Sfenthourakis, Eleftherios Zouros

**Affiliations:** 1 Department of Genetics, Development & Molecular Biology, School of Biology, Aristotle University of Thessaloniki, 54124 Thessaloniki, Greece; 2 Section of Animal Biology, Department of Biology, University of Patras, 26500 Patras, Greece; 3 Section of Zoology and Marine Biology, Dept. of Biology, National and Kapodistrian University of Athens, Panepistimioupolis, Ilissia, 15784 Athens, Greece; 4 Department of Biology and Natural History Museum of Crete, School of Sciences and Engineering, University of Crete, P.O. Box 2208, 71409 Heraklion, Crete Greece; 5 Department of Biological Sciences, University of Cyprus, Panepistimiou Ave. 1, Aglantzia, 2109 Nicosia, Cyprus; 6 Department of Biology, University of Crete, Heraklion, Crete Greece

**Keywords:** Evolution, Greece, Public acceptance, Science communication

## Abstract

Herein we present the recently founded Hellenic Evolutionary Society (HEVOS) that has been recently instituted to promote evolution and scientific thinking among the Greek-speaking public. HEVOS is a timely initiative, given the low levels of acceptance of evolution by Greek society and the almost complete lack of evolution teaching in primary and secondary education in Greece. Herein, the main aims of the Society are presented.

It’s never too late. As of last January (2019), 160 years after the publication of the ‘On the Origin of Species’, Greece has its new Society aiming to promote evolutionary thinking, the Hellenic Evolutionary Society (HEVOS). In a country where the acceptance of evolution seems to be disappointingly low, at least for European standards [1], HEVOS has a lot of work ahead. Of course, we still lack concrete quantitative data on the acceptance of evolution in Greece, and this should be one of the many tasks that HEVOS has to start working on. According to its charter, the scopes of the Society include the collection and dissemination of information on evolution, supporting young researchers, such as PhD students, by various means (e.g. by sponsoring lab visits, field trips and participation in evolution meetings), the organization of symposia, congresses, lectures, open discussions and other fora on evolution-related subjects and topics, the publication—either electronic or hardcopy—of books, leaflets, essays, educational material etc., and the promotion of communication among its members and the society at large, using all available means, such as websites and social networks.

All these notwithstanding, the most important task for HEVOS is to convince the majority of Greek-speaking people that evolution is not ‘just another theory’, but a robust scientific theory, what we might call a ‘fact’ in every-day speech. To do so, HEVOS has to struggle with a broad range of misunderstandings, prejudiced views, ideological constraints, religious beliefs, and opposition by traditional ‘strongholds’ in the Greek society.

Among the most urgent priorities is the introduction of a concise, scientifically-based content on evolution into general education curricula, especially in secondary education. Today, High School students may be fortunate enough to be taught just a brief chapter on evolution, but in reality, most never are. In fact, all biology courses have been significantly restricted in secondary education, so HEVOS has to join forces with all biologists to re-establish biology as a major field in high school curricula and, in doing so, bring in the frontline the light that gives meaning to all biology, which is evolution.

In addition, HEVOS plans to set up a series of public events that will attempt to change how people anticipate or think of evolution, thus promoting general scientific thinking. Furthermore, seeking synergies and collaboration with other similar societies in Europe and other parts of the world, it aims to contribute towards a global acceptance and understanding of evolution.

Many scientists and educators have already expressed a vivid interest to participate in HEVOS, especially after its first public appearance through a press release that denounced a recent attack to evolutionary theory by people in a science-based guise (see the video release at https://www.youtube.com/watch?v=sKYIBc3wHug and HEVOS’s response—in Greek—available at the website of the monthly magazine *Athens Review of Books* https://athensreviewofbooks.com/arxeio/teyxos106/3985-i-prosfati-epithesi-kata-tis-theorias-tis-ekseliksis). Most founding members of HEVOS are established scientists working on a variety of evolution-related fields and affiliated with the majority of major academic and research institutes of Greece, but they also include well-known science journalists and teachers from secondary education. All members of the first elected Board of Directors are going to do their best to convince Greek society and scholars to embrace evolutionary thinking, which is also a tool for a better understanding of many aspects of the living world and human behaviour.

HEVOS needs all the help it can get by anyone sharing its vision of a society whose decisions are based on rational thinking, scientific evidence and a sound understanding of our own position in the biosphere and the universe.

## Conclusions

HEVOS is an ambitious, but timely and necessary, initiative, which we hope will be received with enthusiasm by a large number of scientists and other people that see the necessity of promoting evolutionary thinking among Greek society. For prospective member application and further information on the activities of HEVOS, please visit the Society’s website: https://hevos.nhmc.uoc.gr.

## Data Availability

Not applicable.
